# Neuroinflammation and Infection: Molecular Mechanisms Associated with Dysfunction of Neurovascular Unit

**DOI:** 10.3389/fcimb.2017.00276

**Published:** 2017-06-20

**Authors:** Abolghasem Tohidpour, Andrey V. Morgun, Elizaveta B. Boitsova, Natalia A. Malinovskaya, Galina P. Martynova, Elena D. Khilazheva, Natalia V. Kopylevich, Galina E. Gertsog, Alla B. Salmina

**Affiliations:** ^1^Research Institute of Molecular Medicine and Pathobiochemistry, Krasnoyarsk State Medical University named after Prof. V.F. Voino-YasenetskyKrasnoyarsk, Russia; ^2^Department of Paediatrics, Krasnoyarsk State Medical University named after Prof. V.F. Voino-YasenetskyKrasnoyarsk, Russia; ^3^Department of Children Infectious Diseases, Krasnoyarsk State Medical University named after Prof. V.F. Voino-YasenetskyKrasnoyarsk, Russia

**Keywords:** infectious diseases, blood-brain barrier, immune response, neurodegeneration, brain development

## Abstract

Neuroinflammation is a complex inflammatory process in the central nervous system, which is sought to play an important defensive role against various pathogens, toxins or factors that induce neurodegeneration. The onset of neurodegenerative diseases and various microbial infections are counted as stimuli that can challenge the host immune system and trigger the development of neuroinflammation. The homeostatic nature of neuroinflammation is essential to maintain the neuroplasticity. Neuroinflammation is regulated by the activity of neuronal, glial, and endothelial cells within the neurovascular unit, which serves as a “platform” for the coordinated action of pro- and anti-inflammatory mechanisms. Production of inflammatory mediators (cytokines, chemokines, reactive oxygen species) by brain resident cells or cells migrating from the peripheral blood, results in the impairment of blood-brain barrier integrity, thereby further affecting the course of local inflammation. In this review, we analyzed the most recent data on the central nervous system inflammation and focused on major mechanisms of neurovascular unit dysfunction caused by neuroinflammation and infections.

## Introduction

Upon confronting different stimuli such as infectious diseases, toxins, and traumatic shocks, the host cells present an orchestrated mechanism of actions to maintain the stability of body tissues. The innate immune cells (i.e., macrophages, dendritic, and mast cells) primarily interact with antigens in non-specific pathways and stimulate tissue homeostatic (inflammatory) responses (Medzhitov, 2008). Some infectious agents can trigger intensive tissue inflammatory responses and activate the complement system. The inflammatory responses in the peripheral tissues cause dendritic cells to activate the adaptive immune system and induce some robust responses (i.e., necrosis as a result of phagocytosis). The tuned connection of the central nervous system (CNS)-immune system supports the host immune defense through various pathways such as inducing fever, pain sensitivity, and increasing the sleeping time (Maier et al., 1998).

There are substantial differences between inflammatory responses arising from the CNS and other body tissues. Perhaps the main distinction is the lack of memory T cells (adaptive immunity) in the brain parenchyma. Memory T cells present the specific antigens of invading pathogens and leaving the CNS they enter lymph nodes and initiate cellular immune responses in lymphoid tissue. In the healthy brain, parenchymal T-cells are located in the cerebrospinal fluid (CSF) but in pathological conditions they enter the CNS through the choroid plexus and meningeal veins (Charo and Ransohoff, 2006). These cells leave the CNS through the cribriform plate into the deep cervical lymph nodes (Andres et al., 1987; Goldmann et al., 2006; Ransohoff and Engelhardt, 2012; Louveau et al., 2015). On the other hand, B-cells, another group of adaptive immune cells, can enter the normal brain. However, their quantity and trafficking slightly increase after the outbreak of some pathologies such as AIDS (Anthony et al., 2003). B-cells can differentiate to plasmoblasts and produce antibodies with different functions in the CNS or exert antibody-independent activities such as cytokine production and activation of T-cells (Ransohoff et al., 2015). The brain parenchymal innate immune cells mainly consist of resident myeloid cells, astrocytes, and microglia, which enter the brain and spinal cord parenchyma during the early embryonic period (Sanes and Lichtman, 1999; Del Rio and Feller, 2006). Microglia are the key players in the physiological development of the brain (Schafer et al., 2012). However, other cell populations within the CNS, such as astrocytes, myeloid cells, and dendritic cells also contribute to the functional activities and homeostasis of the brain (Chen and Regehr, 2000; Ransohoff et al., 2015).

**Figure 1:**
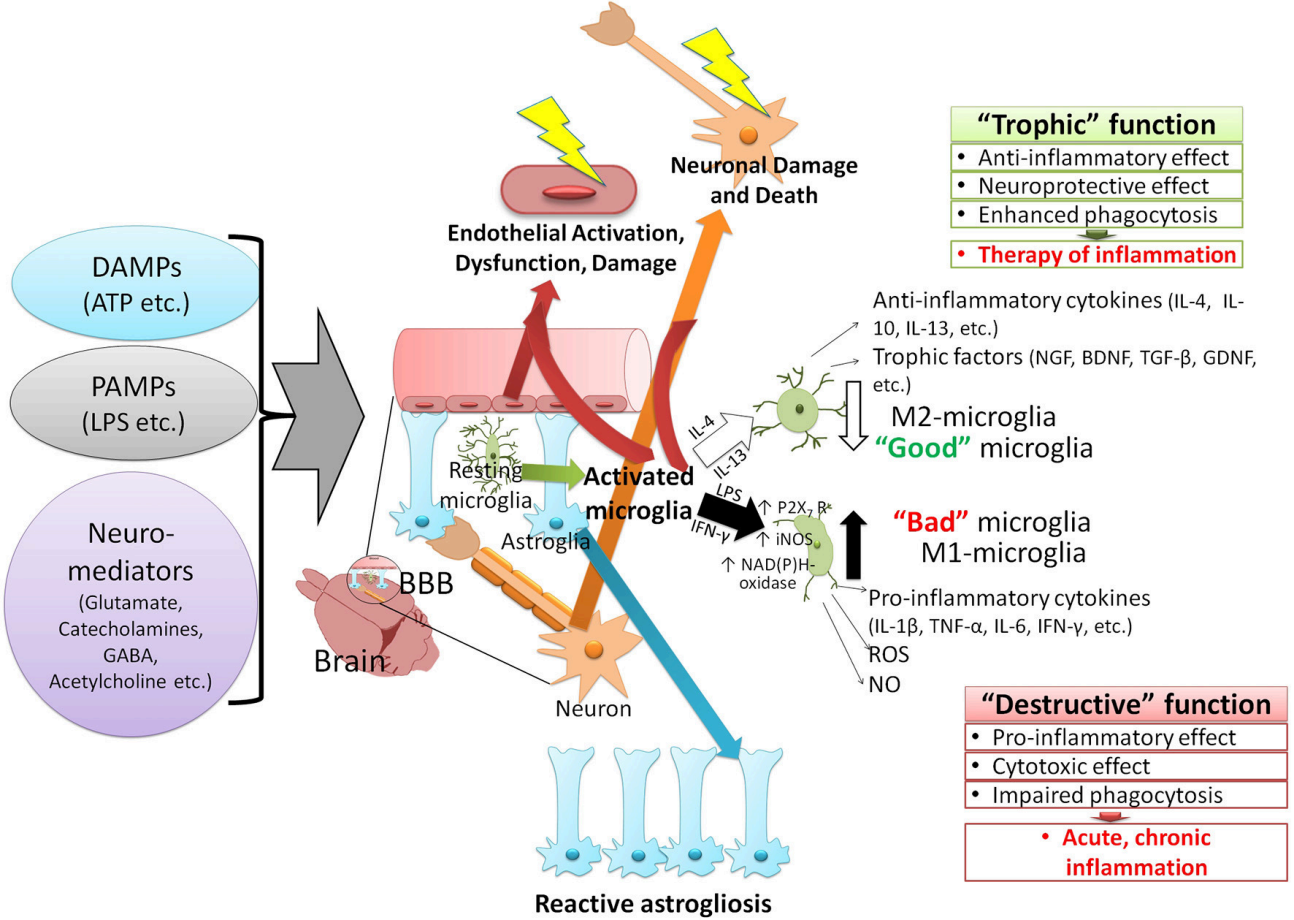
The paradigm of the CNS neuroinflammation. Various factors can activate the immune response of the CNS and induce neuroinflammation. These stimuli are classified into two groups: 1—pathogen-associated molecular patterns (PAMPs), which are produced by the invading microorganisms of the CNS and 2—damage-associated molecular patterns (DAMPs), molecules that are released by host due to onset of traumatic conditions or interaction with some neurotransmitters (i.e., glutamate, GABA, and acetylcholine). The immune responses to the CNS stimuli vary based on the type of stimulation but generally lead to similar outcomes such as immune adaptation, dysfunction, degeneration, and resolution. Activation of the resting microglia and converting them to two distinct phenotypes depends on various cytokines produced by surrounding cells (glia, neurons, migratory immune cells). The release of interleukins 4 and 13 (IL-4, IL-13) gives rise to M1 phenotype (anti-inflammatory) of microglia, which express inflammatory cytokines (interleukin 4, 10, and 13), cell growth factors (i.e., NGF, BDNF, TGF-β, GDNF), and exert anti-inflammatory effects. Interferon-γ (IFN-γ) and the lipopolysaccharide (LPS) of bacteria, on the other hand, activate the M2 phenotype (pro-inflammatory) of microglia. The M2 phenotype is characterized by 1—activation of purinergic receptors P2X7 subtype (activated by ATP, promoting the inflammation and destruction of cells by forming channels and pores), and 2—expression of enzymes which generate reactive oxygen and nitrogen [NAD(P)H-oxidase, iNOS], and trigger the expression of proinflammatory cytokines (IL-1β, TNF-α, IL-6, IFN- γ). Activation of microglia, especially the formation of M2 phenotype exacerbates the damage to BBB (in particular neurons and endothelial cells). Effects of these agents (PAMPs, DAMPs, neuromediators) on astroglia cause their proliferation, activation (reactive astrogliosis), and dysfunction (in particular, increased procoagulant activity and thrombosis). These CNS stimuli also cause endothelial injury, damage, and neuronal death. GABA, γ-aminobutyric acid; IL, Interleukin; NGF, nerve growth factor; BDNF, brain-derived neurotrophic factor; TGF, transforming growth factor; GDNF, glial-derived neurotrophic factors; iNOS, inducible nitric oxide synthase; TNF, tumor necrosis factor; IFN, interferon; ROS, reactive oxygen species; NO, nitric oxide.

It is clear that the impairment of blood-brain barrier (BBB) contributes to neuroinflammation. BBB and activated microglia are components of the neurovascular unit (NVU), and their structural and functional integrity is a major factor affecting the course of neuroinflammation. Migration of immune cells, transport of cytokines and other inflammatory reactions occur due to the increased permeability of BBB and dysfunction of NVU. Therefore, coordinated activity of NVU cells (brain microvessel endothelial cells, pericytes, perivascular astrocytes, neuronal cells, etc.) is necessary to regulate local inflammation in brain tissue. For instance, the integrated neuronal activity and the stimulation of astroglial cells (so-called neuron-astrocyte metabolic coupling) cause the accumulation of extracellular lactate and H^+^ and affect the local inflammatory reactions in the brain. The increased level of neuronal activity also stimulates an inflammatory response in the peripheral tissues—so-called neurogenic inflammation (Figure 1; Roosterman et al., 2006; Chiu et al., 2012). Neurogenic inflammation often occurs due to pain, stress, and epileptic seizures and has a typical degree of similarity to other forms of CNS neuroinflammation (Zochodne et al., 2001; Beggs et al., 2010; Gruber-Schoffnegger et al., 2013).

Taken together, neuroinflammation is a form of inflammatory response within the CNS, which is significantly affected by the status of neuronal activity and BBB permeability. Moderate inflammatory responses can protect the CNS whereas an intensive inflammation aggravates the impairment of tissue homeostasis (Combes et al., 2012; Ransohoff and Brown, 2012; Skaper et al., 2012). In this review, we summarize the current knowledge on the role of BBB/NVU alterations in the development of neuroinflammation, with the emphasis on inflammatory processes caused by infectious diseases. Initially, an overview of physiologic development of NVU and BBB is made and we describe the main factors which modulate the development of neuroinflammation and other neurodegenerative disorders in the CNS. This is followed by discussing the impact of infectious diseases on inducing neuroinflammatory responses in the CNS. Finally, we unfold how neuroinflammation in the CNS is connected with genetic abnormalities of the host.

## NVU/BBB development during the embryonic, fetal, and after-birth periods

BBB formation starts in the uterus and continues during the early postnatal period through several stages as 1-vascularization and angiogenesis (the formation of the choroid plexus and overgrowth of blood vessels); 2-differentiation of cerebral endothelial cells; and 3-the maturation of cellular elements of BBB (Engelhardt and Liebner, 2014).

Vascularization and angiogenesis of BBB happen after the penetration of neuroblasts in the cranial area, where they form the perineural vascular plexus. This is followed by the proliferation of blood vessels from the perineural vascular plexus. The blood vessels grow radially into the neuroectodermal tissue and form multiple small spines that connect with the neighboring vessels. Throughout the prenatal period, vascularization is associated with the formation of a mature spatial structure and a peak of angiogenesis activity that remains stable until the early postnatal period (Ma et al., 2012).

Unlike other tissues, CNS vascularization is exclusively driven by the angiogenesis. Different factors such as vascular endothelial growth factor (VEGF), angiopoietin-1, and sonic hedgehog protein (Shh) change the phenotype of endothelial cells of the perineural vascular plexus to blood vessels that sprout into the neural tube (Lippmann et al., 2012). Differentiation of endothelial cells requires a basal membrane formed by various extracellular matrix proteins (collagen IV, fibronectin, and laminin-1). The coverage of the microvasculature by pericytes purposely determines them as the first neurovascular unit cells to physically interact with endothelial cells (Virgintino et al., 2007). Pericytes, together with neighboring neural progenitor cells and radial glia might also influence the BBB development and induce barrier properties in the brain endothelial cells (Weidenfeller et al., 2007; Daneman et al., 2010b).

During the fetal period, endothelial cells acquire the phenotypic properties of specific tissues and inhabit areas of the developing brain, containing neuroepithelial cells, radial glia, neurons, and neuroblasts (Lippmann et al., 2012). Immunophenotyping of endothelial progenitor cells showed that they express some key antigens markers such as CD31, CD34, and CD45 (Sukmawati and Tanaka, 2015). Mobilization and collection of endothelial progenitor cells in the developing and mature brain are regulated by paracrine and endocrine signals that are produced by BBB. VEGF, Insulin-like growth factor-1 (IGF-1) and angiopoietin-2 are crucial for the mobilization of endothelial progenitor cells. During embryogenesis, endothelial cells retain the important function of regulating neurogenesis by maintaining a population of the brain stem and progenitor cells using growth factors such as brain-derived neurotrophic factor (BDNF), leukemia inhibitory factor (LIF), and Platelet-derived growth factor (PDGF). These processes also occur in the mature brain during the postnatal period (Urban and Guillemot, 2014). In the embryonic brain, radial glial cells, and neuroepithelial cells interact with astrocytes and assist the maturation and maintenance of BBB. Several growth factors and signaling molecules such as angiopoietin-1, cyclic adenosine monophosphate, and basic fibroblast growth factor affect the BBB phenotype *in vitro*. It is clear that the BBB phenotype is influenced by the local microenvironment and is not intrinsic to brain endothelial cells themselves (Lippmann et al., 2013).

Experiments on animal models (rodents) showed that BBB formation begins about 10 days after the embryonic development, followed by the expression of transporter molecules and tight junction proteins. BBB shows transendothelial resistance, which gradually increases during the early postnatal period and corresponds to the formation of full-fledged tight junctions (Siegenthaler et al., 2013). In contrast to animal models, many fundamental features of human BBB development and maintenance remain unclear. After 5 weeks of gestation, the first vessels of the brain start to form as vesicles. During weeks 6–7, these vesicles develop to form a capillary coat in the ventricular zone (Budday et al., 2015). After 15 weeks, the vessels penetrate radially through the nervous tissue and develop into collateral vessels with increased vascular density. During weeks 20–22, the growth of horizontal branches in the lower half of the cortex is detected. This phenomenon is followed by the growth of telencephalon microvessels and the expression of claudin 5 in endothelial cells (Norman and O'Kusky, 1986; Virgintino et al., 2007).

The final step of BBB development is maturation and formation of the NVU. These events are regulated by factors such as VEGF, death receptor 6 (DR6), and tumor necrosis factor receptor superfamily member 19 (TNFRSF19; Obermeier et al., 2013). The neuroectodermal cells, perivascular astrocytes, and pericytes induce the expression of BBB-associated proteins in the brain endothelial cells, which in contact with neurons and glial cells form the BBB (Moretti et al., 2015). BBB maturation in the postnatal period is determined by the formation of stable cell-to-cell interactions within the NVU (Daneman et al., 2010a) and regulated by various humoral factors (i.e., hormones, cytokines, and neurotransmitters).

## BBB alterations in neuroinflammation: molecular targets for inflammation-inducing stimuli

Microglia are the primary components of the CNS innate immune system. They produce cytokines and monitor the integrity of CNS. Microglia comprise about 5–20% of brain glial cell population (Sousa et al., 2017) and are found both in the white and gray matter of the brain and spinal cord. Microglia develop early during the embryogenesis and then migrate to the CNS (Ginhoux et al., 2010). The relatively small turnover of microglia makes them sensitive to the effect of inflammatory stimuli such as trauma, stress, and age (Ajami et al., 2007; Ginhoux et al., 2010). Microglia are crucial for surveying their microenvironment (Davalos et al., 2005; Nimmerjahn et al., 2005) and transporting the inflammatory signals (Dantzer et al., 2008) to the CNS. Activated microglia respond to inflammatory signals by modifying their gene expression at the transcription level and releasing cytokines that assist in recruiting leukocytes to the CNS (Zhou et al., 2006).

The regulation of neuroinflammatory responses is often a result of cooperation between different chemokines [such as chemokine (C-C motif) ligand 2 (CCL2), chemokine (C-C motif) ligand 5 (CCL5), and chemokine (C-X-C motif) ligand 1 (CXCL1)], cytokines (such as IL-6, and TNFα), reactive species of oxygen and secondary messengers (such as prostaglandins). Most of these factors are secreted by activated microglia, which support the synaptic connections and enhance the immunological responses of CNS (Norden and Godbout, 2013; Schafer and Stevens, 2013). Other resident cells of the CNS endothelium and macrophages are also crucial for modulating the inflammatory signals (Dunn et al., 2006). Cytoskeletal rearrangement of activated microglia results in the modification of receptor patterns on their surface, which assist their migration to the source of inflammation (Russo and McGavern, 2015) to represent their macrophage-like activity on the site of injury or infection (Davalos et al., 2005). Therefore, the activated functions of microglia help to defend the CNS against the adverse effects of hostile threats while their over-activation might lead to some neuropsychiatric disorders such as depression (Norden and Godbout, 2013).

Alzheimer's disease (AD) is an example of chronic neuroinflammatory disorder. AD is associated with the activation of microglia, infiltration of the brain tissue with peripheral immune cells, and misfolding and accumulation of several essential proteins (Walter et al., 2007; Sokolova et al., 2009). These phenomena can eventually cause neuronal damage and lead to death (Bucciantini et al., 2002; Sokolova et al., 2009). Unlike in other autoimmune inflammatory diseases, the brain is the initial site of accumulation of soluble β-amyloid and phosphorylated Tau proteins which lead to AD (Van Eldik et al., 2016). Accumulation of β-amyloid causes cell toxicity, which might be counteracted by the expression of transporter molecules in the BBB to transfer the β-amyloid from the brain tissue to the blood. However, this mechanism appears to be affected in AD. Further, progression of neuroinflammation results in deposition of insoluble proteins aggregates, release of alarmins from the affected cells [i.e., HMGB1 (high mobility group box-1)] and elevated permeability of BBB leading to the migration of immune cells to the brain tissue. Alzheimer's type neurodegeneration and inflammatory responses greatly depend on metabolic alterations of neuronal and glial cells (i.e., impairment of neuron-astrocyte metabolic coupling, hypometabolism of glucose, glycolysis, and mitochondria-controlled metabolic changes in microglia) and disruption of calcium homeostasis in neuronal and astroglial cells. Such changes are linked to β-amyloid-induced hypervascularity (extensive angiogenesis) and hyperpermeability of newly-formed cerebral microvessels (Biron et al., 2011). Accumulation of β-amyloid is also related to endothelial dysfunction, vasoconstriction and regional cerebral hypoperfusion of damaged cerebral vessels in AD (Thomas et al., 1996; Niwa et al., 2000; Suter et al., 2002; Townsend et al., 2002; Smith and Greenberg, 2009). The local neuroinflammation in AD stimulates the impairment of BBB/NVU, while the loss of functional and structural integrity in the BBB promotes inflammatory alterations (Salmina et al., 2010).

In addition to the chronic inflammation, CNS can be also affected by the so-called acute neurodegeneration, which is caused by various stimuli such as stroke, head injury, and cerebral or subarachnoid hemorrhage. Some of the markers of acute neurodegeneration are the release of certain chemokines and cytokines (i.e., TNFα, IL-1β, and IL-6), and the activity of microglia. The occurrence of acute neurodegeneration has positive impacts on the coordination of the CNS function to deal with the peripheral injuries or infections and improve the behavioral and physiological responses (Imeri and Opp, 2009). During acute neurodegeneration, migration of the peripheral immune cells to the CNS is not significant, and no adverse effects such as cell atrophy or impairment of the BBB occur. It is therefore suggested that development of acute neurodegeneration assists the optimal local immune responses and provides the microenvironment that is necessary for brain recovery (Tarr et al., 2014). However, acute neuroinflammation can also associate with excessive neuronal injury, BBB leakage, and neurological deficits that hamper neuronal regeneration and recovery.

## Contradictory aspects of neuroinflammation

The close relationship between brain and spine injuries and development of neuroinflammation unveils a cascade of modulating events in affected hosts. CNS injuries activate microglia and astrocytes, release certain chemokines and cytokines and accelerate the migration of peripheral immune cells to the CNS (Werner and Engelhard, 2007). They also cause edema, which is associated with elevated BBB permeability. Moreover, injuries to the CNS trigger post-traumatic symptoms with short-term inflammatory responses that can lead to long-term damages (David and Kroner, 2011; Woodcock and Morganti-Kossmann, 2013). The development of neuroinflammatory responses by the host immune system plays a dual role, which can be harmful or beneficial, depending on the type and extent of the stimulation. Damages to the CNS stimulate the inflammatory and repair responses of microglia and reveal their pro-inflammatory and neuroprotective roles, respectively.

The cytokine-mediated sickness behavior is an example of the positive effect of neuroinflammation. The sickness behavior is induced upon the activation of the immune system by various stimuli carried to the CNS. The corresponding inflammatory signal is processed in the NVU, brain stem, and circumventricular areas of the CNS (Laflamme et al., 1999; Hansen et al., 2001; Ching et al., 2007) which produce cytokines such as IL-1β, TNF-α, and IL-6 (Henry et al., 2009; Chen et al., 2012). These chemical messengers connect the CNS to the immune system and stimulate the development of sickness behavior, which is defined by fever, hypophagia, lethargy, listlessness, and reduced social communications (Dantzer et al., 2008). It is thought that the development of sickness behavior is evolutionarily necessary to counteract against life-threatening antigens and infections (Bluthe et al., 2000; Berg et al., 2004) without further losing the BBB integrity or transfer of peripheral immune cells to the CNS (Dantzer et al., 2008).

Other positive effects of neuroinflammation are immune conditioning (dose-dependent stimulation of the peripheral immune system using bacterial cells; DiSabato et al., 2016), developing the brain plasticity (stimulation of neurogenesis in the neurogenic niches), and assisting the repair process in the brain. On the other hand, some of the major negative impacts of neuroinflammation are injury-related hyperinflammatory responses, which trigger noradrenergic signaling (DiSabato et al., 2016) and activate inflammasomes in the effector cells. These events accelerate the aging process of the brain by activating the immunologically challenged microglia.

Altogether, it can be concluded that the acute neuroinflammation is the positive and beneficial aspect of the CNS immune response whereas the chronic state of neuroinflammation is associated with brain damage and prolonged neurological deficits.

## Association of infectious diseases with neuroinflammation

Activation of local inflammation often starts from the BBB endothelial cells equipped with the molecular machinery to sense bacterial and viral antigens. The first line of defense against microbial invasion comprises the antigenic recognition of a large group of conserved molecular determinants, called pathogen-associated molecular patterns (PAMPs; Hanke and Kielian, 2011) by pattern recognition receptors (PRRs). The PRRs are located on the surface or within the cytoplasm of antigen presenting dendritic cells, macrophages, or other none-immune cells. PRRs activate the innate immune responses, trigger the phagocytic pathways and directly bind to the invading microorganisms. Toll-like receptors (TLRs) are essential PRRs, containing repeats of leucine residues on the N-terminus and a highly conserved C-terminal domain so-called Toll/interleukin (IL)-1 receptor (TIR). Expressed by glial cells and neurons, TLRs are necessary for priming the adaptive immune responses and inducing the release of co-stimulatory molecules and inflammatory cytokines (Hanke and Kielian, 2011). TLRs also recognize a distinct group of host-derived molecules called danger associated molecular patterns (DAMPs), which are released upon the onset of diseases and infections that cause necrosis, apoptosis or tissue damage (Kirschning and Schumann, 2002; Kaisho and Akira, 2004; Kariko et al., 2004; Piccinini and Midwood, 2010; Hanke and Kielian, 2011). The TLR-mediated DAMPs and PAMPs recognition is followed by forming an intracellular molecular machinery (inflammasome) for caspase-dependent processing of the cytokines (IL-1, IL-18). Expression of inflammasomes by the activated glial cells stimulate the brain-leukocyte infiltration (Alfonso-Loeches et al., 2016). In particular, chronically elevated levels of IL-1 in brain tissue was shown to provoke BBB breakdown and neutrophil recruitment (Ferrari et al., 2004). Therefore, measurement of IL-1 and IL-18 levels in the CSF seems like a useful tool to evaluate the severity of the neuroinflammation, i.e., in AD (Wang et al., 2015).

The state of neurological and neurodegenerative diseases can be influenced by peripheral factors such as the gut microbiome (Dinan and Cryan, 2015). Our gastrointestinal tract is perpetually covered with a population of microbial flora (Ley et al., 2006) with the ability to cause significant impacts on the brain by releasing neurotransmitters, hormones, and neuropeptides (Selkrig et al., 2014; Wall et al., 2014). Some examples of these effects are neurodegenerative diseases, depression and autism spectrum disorder (ASD; Putignani, 2012; Mayer et al., 2014; Schroeder and Backhed, 2016; Sharon et al., 2016). Recent findings suggested that human microbiome can significantly affect brain function (Stilling et al., 2014), development (O'Mahony et al., 2017; Tognini, 2017), and neuroinflammation (Rea et al., 2016). The gut microbiota-BBB communication is evident since the gestation period: normal gut flora is required for BBB maturation, establishment of tight junctions, and BBB permeability (in mice; Braniste et al., 2014). Alterations in the composition of normal flora is associated with severe neurological disorders which affect brain development, plasticity, and cause behavioral abnormalities (El Aidy et al., 2014). Parkinson's disease (PD) is a multifactorial neurodegenerative disorder, which is associated with the accumulation of specific amyloid protein, called α-synuclein (α-Syn), a phenomenon that also occurs in other PD-related diseases such as multiple system atrophy (Brettschneider et al., 2015; Sampson et al., 2016). Patients with PD show significant gastric (intestinal) inflammation and majorly suffer from motor deficiencies. Study of a mouse model of Parkinson's disease suggested that the motor circuits dysfunctions are linked to the gastric tract abnormalities and highlighted the crucial role of gut microbial population in triggering the augmentation of α-Syn in PD (Sampson et al., 2016). Host immune responses against infectious diseases are also involved in the pathogenesis of depression and ASD (Miller and Raison, 2016). Several studies have pinpointed the association of aberrant neuroimmune responses with autism and development of depressive behavior (Buehler, 2011; McCusker and Kelley, 2013). In autism, neuroinflammation might occur in the intrauterine period of ontogenesis, and might further progress in the postnatal life in association with impaired angiogenesis and BBB hyperpermeability (Azmitia et al., 2016). In autistic patients, the loss of BBB integrity is linked to the low expression of tight junction proteins and elevated permeability of their intestinal barrier (Fiorentino et al., 2016). These findings suggest that intestinal microflora has a causative role in progression of autistic behavioral abnormalities (Diaz Heijtz, 2016).

One of the most notorious infections of CNS is caused by *Mycobacterium tuberculosis*. Tuberculosis (TB) creates a typical pro-inflammatory response of host immune system (Lee et al., 2009). The TB of CNS is probably the most severe type of tuberculosis and in most cases leads to death. Invasion of the CNS by *M. tuberculosis* is associated with formation of myddosome (see below), disruption of BBB due to cytoskeletal rearrangement in cerebral microvessel endothelial cells (Jain et al., 2006; Cervantes, 2017) and matrix metalloproteinase-mediated degradation of BBB (Green and Friedland, 2007). Tumor necrosis factor (TNF) is a proinflammatory cytokine, which is involved in priming the host immune system against *M. tuberculosis* infection by activating the innate immunity and maintaining the granulomas structure. Investigation of the role of TNF in the immune response against TB of the CNS has shown its protective role. Those findings also proved that neurons are essential sources of TNF production to regulate the immune response against pathogens (Francisco et al., 2015).

Some bacterial infections can develop neuroinflammation by altering the expression of endothelin-1 (ET-1; Freeman et al., 2014). ET-1 is an isoform of endothelin, a short peptide with 21 amino acid residues, which is mainly expressed by endothelial cells. ET-1 is important for maintaining the vascular homeostasis (Agapitov and Haynes, 2002; Schinelli, 2006), vascular tone and inflammation (Speciale et al., 1998; Bouallegue et al., 2007; Kohan et al., 2011). Different types of cells such as neurons, cardiomyocytes, and macrophages produce ET-1 (Freeman et al., 2014). Although ET-1 is mainly a vasoconstrictor, it also acts as a pro-inflammatory cytokine, stimulates the aggregation of platelets and induces the expression of leukocyte adhesion molecules. ET-1 also stimulates the synthesis of inflammatory mediators that cause vascular dysfunction and lead to the progression of diseases and inflammation (Teder and Noble, 2000). Several infectious diseases such as malaria (Dai et al., 2012), infection of *Rickettsia conorii* (Davi et al., 1995), and Chagas disease (Petkova et al., 2001) are associated with ET-1 hyper-expression. These findings indicate the potential role of infectious diseases in developing neuroinflammation by activating ET-1 as a pro-inflammatory cytokine (Freeman et al., 2014). Moreover, expression of ET-1 has been shown to increase in PD (Jain, 2014) and AD (due to the activity of β-amyloid; Palmer et al., 2012). Since overexpression of ET-1 in brain tissue mediates the breakdown of BBB (Zhang et al., 2013), degenerative disorders seen in AD and PD could be, at least partially, caused by the ET-1 hyperexpression.

Taken together, it is crucial to maintain the normal status of body microflora (microbiome), which is necessary for the development of cerebral microvessel endothelial cells. Alterations in the microbiome might provoke inflammation-mediated BBB breakdown or aberrant maturation of newly established cerebral endothelial layer. If so, the association of chronic infections with NVU impairment seen in neurodegenerative or neurodevelopmental diseases is not surprising.

## LPS: a key player in the development of the CNS neuroinflammation

Repeated and minimized contact with infectious agents such as bacterial cells or their constituents can activate the peripheral immune system, thus providing immune protection, in a unique way, which might not involve stimulation of neuroinflammatory responses in the CNS. This phenomenon is called euflammation (Tarr et al., 2014; Liu et al., 2016). Euflammation alters the innate immune system, through regulating the peripheral inflammatory kinetics and controls the receptors that bind to microbial antigens. It also down-regulates the production of pro-inflammatory cytokines, inhibits the activation of brain microglia, and minimizes the development of sickness behavior in animals that received bacterial cells or lipopolysaccharide (LPS; Tarr et al., 2014). Therefore, euflammation can provide some immune protection against bacterial infections and severe toxicity by their endotoxins (Liu et al., 2016).

LPS is a major component of the cell wall structure of gram-negative bacteria and a well-described endotoxin consisting of a polysaccharide chain (varies amongst different gram-negative bacteria) and lipid A (Alexander and Rietschel, 2001). LPS endotoxins are used in modeling bacterial infections and stimulating the infection-associated inflammation via triggering TLR-4, a well-known receptor of LPS (Sandor and Buc, 2005; Rosadini and Kagan, 2017). The interaction of TLR4 with LPS triggers the formation of a macromolecular complex, so called myddosome (Rosadini and Kagan, 2017), including several proteins such as myeloid differentiation primary response gene 88 (MyD88), TIR domain-containing adaptor protein (TIRAP), and interleukin-1 receptor-associated kinase-1 (IRAK). The myddosome complex stimulates the signaling pathways that activate NF-κB (nuclear factor kappa-light-chain-enhancer of activated B cells), activation protein 1 (AP-1), and hyper-expression of several inflammatory genes (Rosadini and Kagan, 2017). TLR4 also regulates other inflammatory responses, such as the release of mediatory microRNAs which sequester LPS-induced pro-inflammatory responses in order to minimize the tissue damage caused by LPS (Molteni et al., 2016).

Nuclear binding domain NOD-like receptors (NLRs) are a main group of PRRs, which detect the bacterial cell wall components and trigger the inflammation. Nucleotide-binding oligomerization domain-containing proteins 1 and 2 (NOD-1 and NOD-2) are two major NLRs that detect bacterial peptidoglycan, induce the release of NF-κB and activate the mitogen-activated protein (MAP) kinase-dependant inflammatory responses (Elinav et al., 2011). Studying the effects of NOD-1 and NOD-2 on brain activity of mice models showed that co-activation of NOD and TLR4 stimulated the peripheral immunity and intensified LPS-activated TLR4 impact on sickness behavior and brain function (Farzi et al., 2015).

Furthermore, recent studies suggested that tolerance to LPS might trigger a late pro-inflammatory response and increase the expression of some anti-inflammatory cytokines that cause inimical injuries to the CNS (Pardon, 2015). Frequent administration of LPS triggers the hyper-expression of IL-1β, TNF-α, and IL-12 in brain but reduces the systematic expression of cytokines. The occurrence of systemic infections increases the potential of brain innate immune cells to develop tolerance and may induce or aggravate the neurodegenerative process due to the damaging effect of cytokine hyper-production (Puntener et al., 2012).

Epilepsy might be an example of an infection-mediated neuroinflammatory response, which contributes to the progression of chronic CNS disorders. Epilepsy is defined by the occurrence of unprovoked brain seizures and affected by a multitude of factors such as genetic background, CNS trauma, and infections (Vezzani and Granata, 2005). Different infections from bacteria, viruses, fungi, parasites, and prions can stimulate the CNS inflammation and induce epileptic seizures (Vezzani et al., 2016). Bacterial LPS can trigger epilepsy in mice and rat models through stimulating the secretion of cytokines, in particular, IL-1β, which is crucial in epileptogenesis (Auvin et al., 2010). Administration of LPS into the peritoneal cavity of rats induces a cyclooxygenase-2 (COX-2) dependent inflammation. COX-2 is a prostanoid-forming enzyme, which is activated during seizures (Rojas et al., 2014). The inducing effect of LPS on COX-2 indicates a higher seizure susceptibility and a more intense oxidative response during the LPS-mediated neuroinflammation (Ho et al., 2015).

Lipoteichoic acid (LTA) is a main constituent of the cell wall structure which is found in gram-positive bacteria, consisting of polyhydroxy alkane repeats. LTA assists with bonding the bacteria to the microvascular endothelial cells of brain (Sheen et al., 2010). Testing the LTA extracts on mice brain revealed that it simulated the release of interferon-γ (IFNγ), IL-6, and other cytokines. LTA was also associated with hyperexpression of circulating corticosterone and diminished expression of tight junction proteins, claudin 5 and occludin, in the brain (Mayerhofer et al., 2017). Upon the onset of bacteriolysis in blood, i.e., as a result of antibiotic therapy, LTA is released and detected by TLR2. LTA-TLR-2 triggers the secretion of inflammatory cytokines such as TNF-α and IL-1b, which ultimately damage BBB (Boveri et al., 2006).

## The impact of infectious diseases on the incidence of neurodegenerative disorders

**Figure 2:**
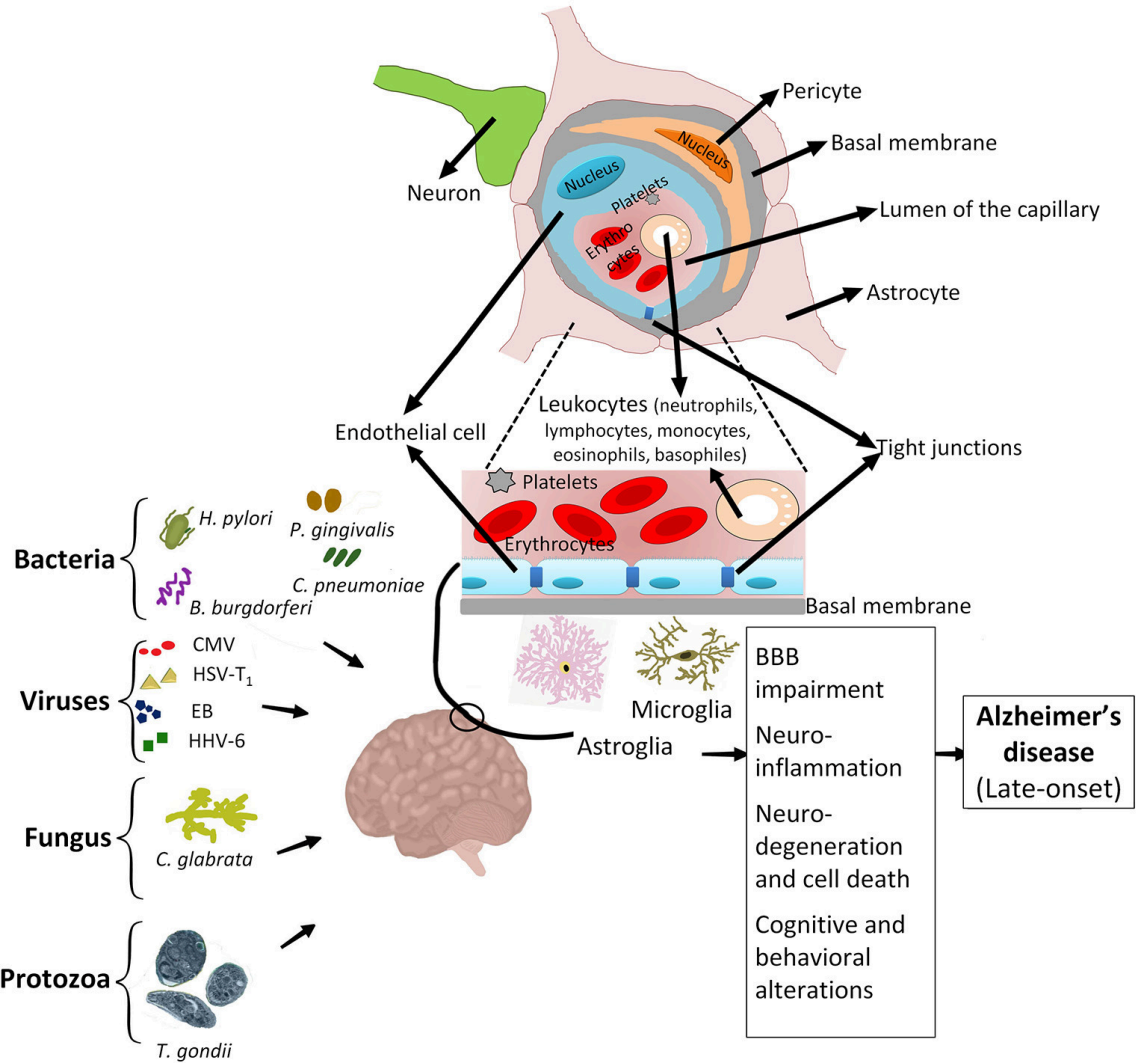
Association of infectious agents with Alzheimer's disease. Chronic infections caused by major infectious agents, i.e., *Helicobacter pylori*, various types of spirochetes, including periodontal pathogen spirochetes and *Borrelia burgdorferi, Porphyromonas gingivalis, Chlamydophila pneumoniae*, Cytomegalovirus, Herpes simplex virus type 1, Epstein-Bar virus, Human herpes virus 6, *Candida glabrata* and *Toxoplasma gondii* are associated with development of AD. Early life exposure to these pathogenic agents can activate the resting microglia and astroglia, trigger the migration of immune cells to the neuro-endothelial tissue, degrade cell-cell tight junctions, and cause the breakdown of BBB. These activities result in development of various side effects such as neuronal damage, neuroinflammation and ultimately predispose the adult patient to develop AD.

As mentioned above, several infectious agents could contribute to neuroinflammation and neurodegeneration. Recently, a group of pathogenic agents including *Borrelia burgdorferi, Porphyromonas gingivalis, Chlamydophila pneumoniae, Helicobacter pylori*, Cytomegalovirus, Herpes simplex virus type 1, Epstein-Bar virus, Human herpes virus 6, *Candida glabrata*, and *Toxoplasma gondii* have been addressed to have a significant effect on the late onset of AD in adults (LOAD; Bu et al., 2015; Lim et al., 2015; Figure 2). The late-age development of AD is purposely due to the activity of infections that initially occurred during the childhood (Khachaturian, 1985). In AD, the association of chronic infections with progressive neurodegeneration was clearly demonstrated by recent studies (Maheshwari and Eslick, 2015). Chronic spirochetal infections can induce β-amyloid accumulation in the brain, cause dementia and reproduce the clinical, pathological, and biological hallmarks of AD (Miklossy, 2011). Thus, infections by spirochete bacteria have significant potentials in developing AD (De Chiara et al., 2012; Hill et al., 2014; Maheshwari and Eslick, 2015). Different species of spirochetes, such as *T. socranskii, T. pectinovorum, T. denticola, T. maltophilum, T. medium, T. amylovorum*, and *Borrelia burgdorferi* (causing Lyme disease) are found in the brain of AD patients (Burgdorfer et al., 1982; Riviere et al., 2002). *T. pallidum* (the causative agent of syphilis) can reside in the brain and cause chronic infection which is associated with inflammation and dementia (Miklossy, 2015). Moreover, infection by some spirochetes such as *B. burgdorferi* could trigger the formation of specific granulovacuolar lesions in neurons and glial cells, which are comparable to those found in AD (Miklossy et al., 2006). AD patients are more vulnerable to infection-mediated cognitive impairment (McManus and Heneka, 2017), particularly in the case of chronic respiratory tract infections (McManus et al., 2014). Since β-amyloid shows antimicrobial properties *in vitro* (Welling et al., 2015; Spitzer et al., 2016), it is postulated that the excessive accumulation of β-amyloid in AD brain could reflect the response of brain neuronal cells to microbial agents. The co-morbidity of neurodegenerative and infectious diseases affects the progression of neurological disorders. For instance, the main cause of death in AD is the onset of infectious diseases such as pneumonia or urinary tract infections (Miklossy, 2015).

*C. pneumoniae* is an intracellular pathogenic bacterium which infects mucosal surfaces and causes respiratory infections such as community-acquired pneumonia (Grayston et al., 1990). *C. pneumoniae* is suggestively associated with other non-respiratory malignancies such as inflammatory arthritis, multiple sclerosis, and AD (Balin et al., 2008). The association of *C. pneumoniae* with pathogenesis of AD has been pinpointed in several studies (Balin et al., 2008; Shima et al., 2010). However, it is not still clear whether *C. pneumoniae* infection actually develops AD as there are some other studies which failed to show this relationship. Perhaps by utilizing a suitable *C. pneumoniae* infection model and applying standard methods which analyse homogenous sample analysis one would be able to elucidate this obscurity (Shima et al., 2010).

*H. pylori* is a gram-negative and spiral shaped bacterium that causes progressive and multistep inflammation of gastric lumen which, can lead to gastric cancer (adenocarcinoma; Tohidpour, 2016). *H. pylori* infection has been linked to high risk of AD in infected patients. Analysis of rat models of *H. pylori* infection showed evidence of memory and spatial learning defects as well as damage to the maturation of hippocampus dendritic spine cells (Wang et al., 2014). Such harmful effects are possibly exerted by soluble components of *H. pylori* surface fractions, which induce formation of Aβ42, a member of β-amyloid (Aβ) family, as main constituents of extracellular senile plaques (SP). Infection by *H. pylori* enhances the activity of γ-secretase, one of two enzymes, which process the amyloid precursor protein to produce mature Aβ, thus increasing levels of Aβ in brain of infected host and contributing to the development of AD (Wang et al., 2014).

## The relationship between viral infections and neuroinflammation in the CNS

The impact of viral infections on neuroinflammation develops through the interaction of CNS-associated immune responses with virulence factors of viruses, which trigger neurodegeneration and apoptosis in the brain (Amor et al., 2010; Czirr and Wyss-Coray, 2012). Viral encephalitis afflicts the CNS with various atrophies, such as loss of memory, acute CNS damage and death. The direct invasion of viruses to CNS can occur through at least five different pathways: (i) by tight junctions of brain microvascular endothelium; (ii) invading the brain microvascular endothelium and penetrating through the basolateral membranes; (iii) transfecting the migrating leukocytes to penetrate and infect the CNS; (iv) migrating through peripheral neuron to the CNS; and (v) infecting the olfactory bulb epithelium and migrating to the CNS (Miner and Diamond, 2016). Microglia and astrocytes are two main types of cells, which are affected during viral infection of the CNS. They produce pro-inflammatory cytokines and various immune-mediator molecules that activate the immune system against viruses (Ransohoff and Perry, 2009; Ransohoff and Brown, 2012; Perry and Teeling, 2013; Phares et al., 2013).

Recognition of viral pathogenic molecular patterns by host PRRs stimulates different signaling pathways and activates transcription factors such as NF-κB and interferon regulatory factor 3 (IRF3). Activated IRF3 induces the expression of interferon β (INF-β), which exerts antiviral activity by inhibiting protein synthesis of viruses. Activated NF-κB triggers the release of some anti-apoptotic proteins and pro-inflammatory cytokines with significant antiviral effects (Santoro et al., 2003; Vercammen et al., 2008). Microglia and astrocytes have been shown to actively respond to both DNA and RNA viruses [i.e., vesicular stomatitis virus (VSV), cytomegaloviruses and Sendai virus] by releasing various types of proinflammatory cytokines and chemokines such as IL-1β, TNF-α, and IL-6 (Furr and Marriott, 2012).

Viral infections also contribute to the development of AD. Viruses such as cytomegalovirus, herpesviridae, and herpes simplex type 1 (HSV-1) can escape the immune system and activate innate and adaptive immune responses via stimulating hyper-expression of pro-inflammatory cytokines with their DNA or RNA (Harris and Harris, 2015). Viral infections either directly or indirectly contribute to AD pathogenesis through various pathways such as increasing the concentration of amyloids, phosphorylating certain neuronal proteins and afflicting neurons with injury. For instance, infections with cytomegaloviruses generate a systematic population of pro-inflammatory cytokines which can pass BBB, trigger the CNS neurodegeneration and lead to AD (Lim et al., 2015). HSV-1 is usually found in the brain of infected adult hosts (Wozniak et al., 2005) and is thought to be involved in development of AD in this group of patients (Agostini et al., 2014). Primary infection with HSV-1 leads to persistent infection of sensory ganglion cells that belong to the peripheral nervous system. Various factors lead to activation of HSV-1 from the latency phase, such as stress and ultraviolet radiation (Itzhaki, 2014). Re-activated HSV-1 affects the hippocampus as well as the frontal and temporal cortices of brain, which are also being affected in AD patients' brains. The immune system is also able to reactivate HSV-1 from latency. However, results of recent experiments have suggested a protective role of humoral immunity against the damaging effect of HSV-1 in the brain of infected patients (Mancuso et al., 2014).

The ability of human immunodeficiency virus (HIV) type 1 to invade and infect the CNS is noteworthy. HIV can penetrate the CNS some weeks after the infection and induce a neuroinflammation pathway, which leads to the CNS injury (Schacker et al., 1996; Pilcher et al., 2001). It is thought that the CNS and the cerebrospinal fluid are potential niches for replication of HIV. The occurrence of genetic mutations in HIV genome leads to the evolution of HIV strains that evolve during early stages of infection and cause CNS inflammation and neurogenic damages (Dahiya et al., 2013). Although the current strategies of anti-viral therapy have drastically reduced neurocognitive impairments caused by HIV, the overall occurrence of such injuries has increased. This increase is linked to the difficulties of accessing the anti-HIV treatments in developing countries. Previous experiments revealed that brain microvascular endothelial cells are crucial for the immunity of the CNS against HIV-mediated neuroinflammation. These cells recognize HIV molecular determinants using TLR3, release antiviral compounds such as INF-β and trigger the phosphorylation of IRF3 and IRF7, activities of which control the interferon signaling pathways and confer immune defense against HIV infection (Li et al., 2013).

The neuroinflammatory disorders induced by West Nile virus (WNV) is another example of a viral infection-mediated CNS neurodegeneration. WNV is a mosquito-borne RNA virus belonging to flaviviruses (Lindenbach and Rice, 2003). WNV is a major pathogen of the CNS neurons and causes viral encephalitis. Microglia and astrocytes of CNS are both infected with WNV and develop apoptosis (Yang et al., 2002; Chambers and Diamond, 2003). A large number of viral proteins, such as envelope and capsid proteins are involved in the neuropathogenesis of WNV (Beasley et al., 2002, 2005; Lee and Lobigs, 2002). Amongst these, capsid proteins are the main viral factors causing neuroinflammation, neurotoxic effects, and apoptosis (Yang et al., 2002). Upon infection with WNV, several PRRs including MDA5 (melanoma differentiation-associated protein 5), TLR3, TLR7, and RIG-I (retinoic acid-inducible gene 1) determine the molecular patterns of WNV. This recognition leads to hyperexpression of inflammatory cytokines such as IL-1 and TNF-α, which inhibit viral replication, enhance the presentation of viral antigens and increase the migration of leukocytes (to eradicate the WNVs from the CNS; Daniels et al., 2014). Activation of PRRs by WNV also triggers the expression of IFN-α and IFN-β (type I INFs), which inhibit viral replication (Samuel and Diamond, 2005) and enhance the capability of the adaptive immune system. *In vitro* studies on BBB models showed that the antiviral activities of type I IFNs regulate the permeability of brain endothelial cells and thus reduce viral movements across the BBB (Daniels et al., 2014).

Two other members of flavivirus family, Zika virus (ZIKV) and Dengue virus are also linked to neuroinflammation of the CNS (Tsai et al., 2016; Roach and Alcendor, 2017). ZIKV, normally causes limited infections with symptoms such as fever, headaches, and conjunctivitis (Lum et al., 2017). However, ZIKV is also associated with brain microcephaly and ocular defects in infants, which occur during the course of pregnancy (Roach and Alcendor, 2017). Following recognition by TLR-3, infection of fetal brain cells with ZIKV impairs the neurosphere and brain organoid growth. ZIKV infection has been also found in Microglia and was shown to increase the level of cytokines such as IL-6, TNF-α, and IL-1β (Lum et al., 2017). Same as WNV and ZIKV, Dengue is also a mosquito-borne RNA flavivirus. Globally, ~2.5 billion people are at risk of infection by Dengue virus (DENV; Guabiraba and Ryffel, 2014). DENV infects around 400 million people each year causing symptoms such as fever, headache and rashes, which are referred as dengue fever stage. A minor proportion of patients who show the symptoms of the dengue fever further develop an acute form of disease called severe dengue haemorrhagic fever (DHF)/and shock syndrome (DSS). Some symptoms of DHF/DSS include gastrointestinal bleeding, renal/hepatic failure and hemorrhage which are determined by the interaction of viral virulence factors with components of host immune system. Strikingly, patients with DHF/DSS are very prone to neurological defects and CNS abnormalities. DENV can invade the CNS by transmitting though BBB and induce encephalitis (Tsai et al., 2016).

Another example of a virus, which also causes encephalitis, is Chikungunya virus (CHIKV). CHIKV is a member of encephalitogenic RNA viruses and is transmitted by mosquito bites (Long et al., 2013; Gerardin et al., 2016). The infection of CHIKV causes debilitating rheumatic diseases and inflammatory disorders which are generally non-fatal. However, in rare cases CHIKV infection can cause neurodegenerative disorders such as meningoencephalitis, myelitis, and Guillain-Barre syndrome (Gerardin et al., 2016; Brizzi, 2017).

Taken together, the onset of viral infections in the CNS can induce neuroinflammation in the glial cells of NVU (Allen et al., 2009; Kuang et al., 2009) and activate host immune responses against the invading viral pathogens. Such immune responses might eventually result in elevated local cytokines/chemokines concentrations and loss of BBB integrity.

## Role of host factors and genetic alterations in neuroinflammation

In addition to infectious agents and injuries, genetic mutations can also induce neuroinflammation. Genetic studies have elucidated the molecular mechanisms underlying the etiology and pathogenesis of various neurodegenerative disorders. Table 1 shows a list of some major immune genes whose mutations significantly induce different neurodegenerative diseases (Table 1).

**Table 1:** Immune genes associated with development of neurodegenerative diseases such as AD, encephalitis, and meningitis.

**Gene/protein**	**Localization**	**Function/activity site**	**Disease (references)**
TREM2 (TREM-2; Trem2a; Trem2b; Trem2c) (rs75932628-T)	Chr 6: 41.16–41.16 Mb	Stimulation of the expression of inflammatory cytokines	Alzheimer's disease (Guerreiro et al., 2013; Jiang et al., 2013; Jonsson et al., 2013; Pottier et al., 2013)
APOE (AD2; LPG; APO-E; ApoE4; LDLCQ5)	Chr 19	Regulation of β-amyloid aggregation in the brain	Alzheimer's disease (Liu et al., 2013)
Genetic factor I (FI)	I322T and D506V	Complement factor I, A serine protease, regulating the immune response alternative pathway activation	Recurrent aseptic meningo-encephalitis (Haerynck et al., 2013)
CARD8 (NDPP; DACAR; DAKAR; NDPP1; TUCAN; CARDINAL) (rs2043211)	Chr 19: 48.18–48.26 Mb	An adaptor molecule, regulating apoptosis, NF-κB, and CASP1-dependent IL-1β	Pneumococcal meningitis (Geldhoff et al., 2013)
NLRP1 (NAC; CARD7; CIDED; NALP1; SLEV1; DEFCAP; PP1044; VAMAS1; CLR17.1; DEFCAP-L/S) (rs11621270)	Chr 19: 48.18–48.26 Mb	Inducing apoptosis	Pneumococcal meningitis (Geldhoff et al., 2013)
NFKBIE (IKBE) (rs3138053)	Chr 6: 44.26–44.27 Mb	Inhibition of NF-κB-mediated cellular hyper-expression	Pneumococcal meningitis (Lundbo et al., 2016)
MBL2 (MBL; MBP; MBP1; MBPD; MBL2D; MBP-C; COLEC1; HSMBPC)	10q21.1	Member of the innate immune system. Activation of the classical complement pathway	Meningococcal disease (Lundbo et al., 2015)
LMP7 (JMP; ALDD; LMP7; NKJO; D6S216; PSMB5i; RING10; D6S216E)	Chr 6: 32.84–32.84 Mb	Low molecular weight proteasome, included in the class II major histocompatibility complex.	LCMV-induced meningitis (Mundt et al., 2016)

Acute hemorrhagic leukoencephalitis (AHLE) is a type of acute disseminated encephalitis that often leads to death. The etiology of AHLE is thought to be associated with upper respiratory infections, mumps, and infection by *Mycoplasma pneumoniae*. The deficit of complement factor I (CFI) is frequently associated with recurrent pyogenic infections such as meningitis and meningoencephalitis (Floret et al., 1991; Leitao et al., 1997). CFI is a regulator of the complement alternative pathway and a major complement inhibitor. Complete deficiency of CFI results in secondary complement deficiency due to uncontrolled and spontaneous alternative pathway activation, and leads to hyper-susceptibility to infections (Nilsson et al., 2009). Broderick and co-workers described two pediatric AHLE patients of Filipino descent with partial CFI deficiency. They showed that the primary site of inflammation was in the CNS and reported symptoms such as headache, hallucinations, and reduced pupillary reaction to light. Physical examination of other patients showed that they had difficulties with speaking and suffered from fatigue. The authors identified two novel missense mutations in CFI by sequencing 13 exons of CFI and suggested that infections may trigger an uncontrolled activation of complement in brain parenchyma of predisposed individuals, leading to severe neuroinflammation and demyelination (Broderick et al., 2013). Moreover, Haerynck and co-workers studied a rare deficiency of CFI in a patient with relapsing inflammatory-mediated meningoencephalitis. They described the case of a 16-year-old patient having headaches, nausea, vomiting, neck stiffness, diplopia, and lethargy as recurrent episodes of acute aseptic meningoencephalitis. MRI of brain further approved the evidence of meningoencephalitis. Mutation analysis of the complement factor I gene showed two heterozygous mutations (I322T and D506V) that resulted in a complete CFI deficiency due to a functional CFI defect (Haerynck et al., 2013).

Clinical studies on the role of genetic abnormalities in pneumococcal meningitis showed the association of meningitis with polymorphisms in the inflammasome genes encoding caspase recruitment domain family member 8 (CARD8; SNP ID: rs2043211) and NLR family pyrin domain containing 1 (NLRP1; SNP ID: rs11621270). Genetic variations possibly influence inflammasome genes and alter the activation threshold of inflammatory responses (Geldhoff et al., 2013). Furthermore, sequencing the coding regions of 46 innate immune genes from 435 patients, Ferwerda and co-workers showed that immune susceptibility to pneumococcal meningitis is related to variations in several genes encoding the CARD8, CXCL1, NOD-2, and interleukin-1 receptor-associated kinase 4 (IRAK4; Ferwerda et al., 2016). Lundbo and co-workers also found the association of pneumococcal meningitis with the polymorphism of nuclear factor of kappa light polypeptide gene enhancer in B-cells inhibitor (NFKBIE) and that the increased risk of invasive pneumococcal disease (IPD) was in the heterozygosity meningitis group for NFKBIE (Lundbo et al., 2016). Taken together, it is noteworthy to consider the impact of genetic variations and genetic abnormalities on function of certain genes, which are responsible for activity, protection, and homeostasis of NVU and the CNS. The onset of malignant mutations that disrupt the physiological functions of such genes can predispose the host to develop severe neurodegenerative disorders and/or manipulate the ability of immune system to defend against infectious agents invading the CNS.

## Concluding remarks

Neuroinflammation in the CNS has two facets: one beneficial and the other destructive. It is not yet clear whether the onset of neuroinflammation is entirely useful or causes further damages. Moreover, the genetic background of host plays a critical role in predisposing the CNS to various neuroinflammatory responses and affecting the ability of the CNS to prevent neuropsychiatric and neurodegenerative disorders.

Microbial infections can trigger the CNS-associated immune responses and cause neurodegenerative and neurodevelopmental disorders. It is well-known that there is a complicated relationship between the host normal flora and the CNS. Majority of our understanding of the microbiome effects on the CNS homeostasis or disorders are derived from the microbiome-gut-brain axis. However, microbiome from other body niches might also be able to de-regulate the CNS and induce neuroinflammatory disorders. For instance, recent studies have found the presence of RNA of α-proteobacteria in human brain (Branton et al., 2013). Some α-proteobacteria are known as pathogen of human, such as *Rickettsia conorii, Rickettsia rickettsii*, and *Delftia acidovorans* (in compromised patients) and/or have been isolated from cerebrospinal fluid (*D. acidovorans*; Pedersen et al., 1970). It is therefore quite possible that such niche-specific microbiomes can directly engage components of host immune system (such as TLRs or NODs to activate brain immune signaling) and trigger neuroinflammation. Therefore, it seems crucial to further determine the effect of non-gut microbiome on homeostasis of the CNS and trafficking of immune constituents. It is practical to neutralize the severity of the CNS inflammation by antibiotic therapy of infections or eradication of inflammation-stimulating effector cells. However, these strategies are unable to guarantee lessening the excessive disadvantages of neuroinflammation or prevent psychological disorders such as mood or other degenerative diseases (Xanthos and Sandkuhler, 2014). Alterations in normal composition of gut microflora can trigger some adverse brain disorders such as activation of hypothalamic pituitary adrenal (HPA) axis (Rea et al., 2016), acute brain ischemia (Singh et al., 2016), and neurodegeneration (Minter et al., 2016). BBB serves as an essential mediator of the CNS-microbiome interactions. In this context, deciphering the mechanisms by which BBB is involved in pathogenesis of the CNS neuroinflammation sounds very important. It is therefore pivotal to further study the close relationship of host normal microbiome(s) and the CNS. This would provide a better understanding of the CNS-immune system interactions to improve the treatment of the CNS injuries and reduce the CNS susceptibility to infectious agents.

## Author contributions

AT and AS wrote and edited the manuscript, and designed Figures 1, 2 and Table 1. AM and NM wrote the manuscript and designed Figures 1, 2. EB wrote the manuscript and designed Table 1. NK, EK, GM, and GG wrote the manuscript.

### Conflict of interest statement

The authors declare that the research was conducted in the absence of any commercial or financial relationships that could be construed as a potential conflict of interest.
